# Macrotroponin Complex as a Cause for Cardiac Troponin Increase after COVID-19 Vaccination and Infection

**DOI:** 10.1093/clinchem/hvac100

**Published:** 2022-07-27

**Authors:** Anda Bularga, Ellen Oskoui, Takeshi Fujisawa, Sara Jenks, Rachel Sutherland, Fred S Apple, Ola Hammarsten, Nicholas L Mills

**Affiliations:** BHF Centre for Cardiovascular Science, University of Edinburgh, Edinburgh, UK; BHF Centre for Cardiovascular Science, University of Edinburgh, Edinburgh, UK; School of Biological Sciences, University of Edinburgh, Edinburgh, UK; BHF Centre for Cardiovascular Science, University of Edinburgh, Edinburgh, UK; Department Clinical Biochemistry, Royal Infirmary of Edinburgh, Edinburgh, UK; Department of Acute Medicine, Royal Infirmary of Edinburgh, Edinburgh, UK; Departments of Laboratory Medicine and Pathology, Hennepin Healthcare/Hennepin County Medical Center and University of Minnesota, Minneapolis, MN, USA; Department of Clinical Chemistry and Transfusion Medicine, Institute of Biomedicine, The Sahlgrenska Academy at the University of Gothenburg, Gothenburg, Sweden; BHF Centre for Cardiovascular Science, University of Edinburgh, Edinburgh, UK; Usher Institute, University of Edinburgh, Edinburgh, UK

**Keywords:** High-sensitivity cardiac troponin, mRNA SARS-CoV-2 vaccine, COVID-19

## Description of Cases

Patient 1 was a 28-year-old Caucasian woman who presented with a 4-day history of chest pain to the same day emergency care unit at the Western General Hospital, Edinburgh, Scotland. The patient had received a second dose of the mRNA-1273 (Moderna) vaccine for COVID-19 five days previously. After vaccination, she felt unwell with fever and dry cough. Shortly after, she developed chest pain. The patient had no background medical history and her clinical examination was unremarkable.

Patient 2 was a 29-year-old Caucasian man who presented with chest pain to the Sahlgrenska University Hospital, Gothenburg, Sweden. The patient had received a second dose of the mRNA-1273 (Moderna) vaccine 3 weeks prior to admission. He described a similar episode of chest discomfort a few days after vaccination. The patient had a history of asthma but was not receiving regular medication. His clinical examination was normal.

Patient 3 was a 19-year-old Caucasian man who had COVID-19 confirmed using a reverse transcription-PCR test. He experienced mild breathlessness and headache and was generally unwell. Six weeks following confirmed COVID-19 infection, he developed chest pain, which prompted admission to the Sahlgrenska University Hospital, Gothenburg, Sweden. The patient had a history of asthma. The clinical examination was normal.

## Investigations

High-sensitivity cardiac troponin (hs-cTn) testing was performed at presentation, repeated 3 or 6 h later, and on follow-up. hs-cTnI was measured on Abbott ARCHITECT*_STAT_* (i2000) or Abbott Alinity (ci), and hs-cTnT was measured on the Roche Diagnostics Elecsys (Cobas e801) platforms. The limit of detection for hs-cTnI ranges from 1.2 to 1.9 ng/L, and sex-specific 99th percentiles are 16 ng/L (females) and 34 ng/L (males). The limit of detection for hs-cTnT is 3 ng/L,and sex-specific 99th percentiles are 9 ng/L (females) and 16 ng/L (males).

In all 3 patients, hs-cTnI concentrations were increased at 31 ng/L, 750 ng/L, and 280 000 ng/L on presentation, but the electrocardiogram was normal, with no ST-segment or T-wave abnormalities (Table 1). Admission laboratory investigations identified normal hematology for all patients.

**Table 1:** Clinical characteristics of patients with cardiac troponin assay interference in the context of COVID-19 infection or vaccination.

	Patient 1	Patient 2	Patient 3
Age, years	29	28	19
Sex	Female	Male	Male
Prior cardiac history	None	None	None
COVID-19 infection	No	No	Yes
Immunization against COVID-19			
Vaccine type	mRNA-1273 (Moderna)	mRNA-1273 (Moderna)	—
Vaccine dose	Second dose	Second dose	—
Symptoms			
Primary presenting symptom	Chest pain	Chest pain	Chest pain
Other symptoms	Cough	Palpitations	Shortness of breath
Laboratory information			
hs-cTnI assay platform	Abbott ARCHITECT*_STAT_* (i2000)	Abbott Alinity (ci)	Abbott Alinity (ci)
hs-cTnT assay platform	Roche Elecsys (Cobas e801)	Roche Elecsys (Cobas e801)	Roche Elecsys (Cobas e801)
Laboratory investigation results			
hs-cTnI, ng/L (presentation)	31	750	280 000
hs-cTnT, ng/L (presentation)	4	33	11
hs-cTnI, ng/L (serial)	37	770	180 000
hs-cTnT, ng/L (serial)	5	—	6
hs-cTnI, ng/L (follow-up)	48	696	93 000
hs-cTnT, ng/L (follow-up)	5	—	10
Macrotroponin complex analysis			
Macrotroponin complex	IgG to hs-cTnI	IgG to hs-cTnI and to hs-cTnT	IgG and IgM to hs-cTnI
hs-cTnI concentration (ng/L) prior to immune complex removal*	58	860	180 000
hs-cTnI concentration (ng/L) after immune complex removal*	10	5	13
hs-cTnI recovery, **%**	16	3	<1
hs-cTnT concentration (ng/L) prior to immune complex removal	<3	33	11
hs-cTnT concentration (ng/L) after immune complex removal	<3	6	11
hs-cTnT recovery, **%**	—	3	—
Macrotroponin complex molecular weight, kDa	200	200	500
Suspected clinical diagnosis	Vaccine-associated myocarditis	Vaccine-associated myocarditis	COVID-19 myocarditis
Clinical investigation findings			
Electrocardiogram	Normal	Normal	Normal
Chest X-ray	Normal	Not performed	Normal
Echocardiogram	Normal	Restrictive cardiomyopathy	Normal
Coronary angiography	Not performed	Not performed	Not performed
Cardiac magnetic resonance imaging	Normal	Restrictive cardiomyopathy	Normal
Myocardial biopsy	Not performed	Restrictive cardiomyopathy	Not performed
Final clinical diagnosis	Musculoskeletal chest pain	Incidental finding of restrictive cardiomyopathy	Musculoskeletal chest pain

Serial measures of hs-cTnI concentrations were 36 ng/L and 37 ng/L for patient 1, 860 ng/L and 770 ng/L for patient 2, and 280 000 ng/L and 180 000 ng/L for patient 3 (Table 1). All patients underwent echocardiography. For patients 1 and 3, this demonstrated normal cardiac size and function with no pericardial or valve disease. Patient 2 had enlarged atria and findings suggestive of a restrictive left ventricular filling pattern. Patients 2 and 3 underwent cardiac magnetic resonance imaging, which was normal in patient 3, but demonstrated reduced ejection fraction at 54% in patient 2. As a result, this patient had an endomyocardial biopsy, which identified enlarged myocytes consistent with a restrictive cardiomyopathy and ruled out myocarditis.

## Cardiac Troponin Immunoassay Interference

Stored surplus sample was tested using a hs-cTnT assay, which returned normal values of 4 ng/L and 8 ng/L for patients 1 and 3, respectively. Patient 2 had an increased hs-cTnT concentration of 33 ng/L, discordant with the hs-cTnI value of 860 ng/L (Table 1).

To test for immunoglobulin-bound cTn complexes (macrotroponins) or interference due to heterophilic antibodies, IgG, IgA, or IgM were removed using affinity chromatography by treating plasma with a protein G column (HiTrap, Protein G HP, GE Healthcare) (1), anti-IgA (CaptureSelect*®* IgA affinity matrix, Thermo Scientific), or anti-IgM (Poros*®*CaptureSelect® IgM affinity matrix, Thermo Scientific) resins in a spin column format using Bio-Rad minicolumn. Macrotroponin is retained on the affinity resin resulting in a lower recovery of cTn. In acute myocardial infarction, recovery is 100% (2). The native molecular weight of cTnI was analyzed using 18% to 42% sucrose gradient ultracentrifugation. Simultaneous centrifugation of standard marker proteins allows estimation of sedimentation coefficients to determine the molecular weight of the protein of interest (3). Free cTnI has a molecular weight of around 20 kDa whereas the molecular weight of macrotroponin complex is around 200 kDa allowing analysis of both the free and the bound fraction (2).

For patient 1, hs-cTnI concentrations decreased from 58 ng/L to 10 ng/L, with a recovery of 16% following removal of IgG. In patient 2, an IgG macrotroponin complex affecting both hs-cTnI and hs-cTnT assays was detected with hs-cTnI concentrations, decreasing from 860 ng/L to 5 ng/L, and hs-cTnT concentrations, decreasing from 33 ng/L to 6 ng/L, with a recovery of 3% in both cases. For patient 3, a combined IgG and IgM macrotroponin complex affecting the hs-cTnI assay was identified with a decrease in hs-cTnI from 180 000 ng/L to 13 ng/L and a recovery of <1%. In patients 1 and 3, hs-cTnT concentrations were too low to be analyzed by ultracentrifugation.

## Follow-Up

Patient 1 returned for follow-up 6 weeks after admission. Her symptoms had subsided, and on further questioning, she described recurrent chest pains that were associated with movement. The pain resolved with simple analgesia. As previously, hs-cTnI concentrations were increased at 48 ng/L, but hs-cTnT concentrations were within the reference interval at 5 ng/L (Table 1). Patient 2 returned for ongoing investigations, and hs-cTnI remained increased on serial testing at 696 ng/L, although his symptoms had resolved. Patient 3 was followed up in the ambulatory clinic and reported no further complaints of chest pain or breathlessness, but hs-cTnI concentrations remained increased at 93 000 ng/L.

## Discussion

While multiple cases of myocarditis have been reported following COVID-19 infection and associated with mRNA vaccination in young adults (4), the cellular and immune mechanisms responsible for these observations are unclear. Although no cases of myocarditis were observed in the randomized, placebo-controlled trials of COVID-19 vaccines, this condition is uncommon, and it is not surprising that safety concerns only appeared during postmarket authorization surveillance. Indeed, myocarditis has been reported as an adverse event with other vaccines, for example, after live vaccinia virus vaccine for smallpox (5).

Establishing the diagnosis of myocarditis may be challenging. In practice, this is based on the detection of an increased cTn, with or without evidence of new impairment of ventricular function, and by the exclusion of other causes of myocardial injury (6). In our case studies, the temporal relationship between the onset of chest pain and mRNA-1273 vaccine or recent COVID-19 infection raised clinical suspicion of myocarditis. However, in all patients, the electrocardiogram was unremarkable, and the findings on imaging with echocardiography and cardiac magnetic resonance imaging did not support the diagnosis. While the sensitivity of echocardiography is limited, one would have expected to identify myocardial edema or patchy late gadolinium enhancement on magnetic resonance imaging or lymphocytic infiltration and active myocyte necrosis on cardiac biopsy if cTn increases were due to acute myocarditis. Furthermore, cTn concentrations were unchanged on serial measurement whereas one would expect a rise and/or fall in concentrations consistent with the acute nature of this condition. Given these findings in otherwise healthy young adults, hs-cTnI immunoassay interference was suspected. Measurement of hs-cTnT demonstrated discordance between hs-cTnI and hs-cTnT concentrations.

False-positive cTn increases due to immunoassay interference have been described in many conditions but not in the context of COVID-19 infection or following vaccination. It is estimated that analytical false positives for cTn occur in approximately 1 in 1000 patients (7). Multiple causes for assay interference have been identified, such as heterophilic antibodies, rheumatoid factor, macrotroponin complex, and human antianimal antibodies in response to monoclonal antibodies used in production of therapeutic agents or vaccination (7). In addition, increases in hs-cTnT concentrations can be associated with reexpression of cTn in skeletal muscle in the absence of cardiac involvement (8). Clinicians should be aware of the possibility of immunoassay interference, particularly if there is discordance between cTn concentrations and the clinical presentation or other investigation results.

In view of the temporal association between COVID-19 vaccine administration or infection and the detection of immunoassay interference, we hypothesize that increased hs-cTn concentrations could be due to interference caused by the immune response to mRNA-1273 vaccination or COVID-19 infection. The plausibility of our hypothesis is supported directly by the identification of macrotroponin complex in all 3 patients and indirectly by a similar observation of interference using an immunoassay for HIV in 2 patients with COVID-19 infection (9). The COVID-19 mRNA vaccines elicit a strong immune response with increased plasma IgA, IgM, and IgG activity and monoclonal antibodies against SARS-CoV-2 spike protein and the receptor-binding domain (10). Immunoglobulins can bind multiple proteins in circulation with the resulting complexes cleared in the lymphatic system. Clearance of the bound protein is delayed resulting in increased plasma concentrations. The phenomenon is well known for prolactin, called macroprolactin, but also occurs for cTn, especially hs-cTnI, possibly because it is more immunogenic compared to hs-cTnT, at least in animal models (7). The hs-cTnI antibodies most often form long-lived antibody complexes resulting in false-positive results (3). cTn increases due to macrotroponin complex do not increase cardiovascular risk; however, they may result in misdiagnosis, overinvestigation, and a risk of harm from invasive procedures, such as cardiac biopsy.

When assessing patients with suspected acute and long-term cardiac complications following COVID-19 infection or vaccination, clinicians should be aware of the potential for false-positive cTn increases due to assay interference. This may have important implications on downstream testing, resource utilization, and, importantly, patient well-being.

**Questions to Consider**

1. What are the causes of cTn assay interference?
2. What is the process of testing for immunoglobulin-bound cTn complexes or macrotroponins when assay interference is suspected?
3. When should the possibility of assay interference be considered in clinical practice?

**Points to Remember**

- Analytical false-positive cTn results occur in approximately 1 in 1000 patients and are often associated with heterophilic antibodies, with immunoglobulin-bound cTn complexes or macrotroponins increasingly recognized.
- Episodes of acute myocarditis temporally related to COVID-19 vaccination or arising during COVID-19 infection have been reported and screening for cardiac involvement is increasingly performed in clinical practice.
- Clinicians should be aware of the potential for cTn immunoassay interference in the clinical setting of suspected acute or long-term COVID-19 vaccine or infection associated myocarditis or cardiac complications as misdiagnosis may have important implications for downstream testing and patient well-being.

## Employment or Leadership

F.S. Apple, *Clinical Chemistry*, AACC.

## Consultant or Advisory Role

N.L. Mills has consulted for Abbott Diagnostics, Roche Diagnostics, Siemens Healthineers, and LumiraDx. F.S. Apple served on advisory board of Siemens Healthineers and Qorvo Biotechnology.

## Stock Ownership

None declared.

## Honoraria

N.L. Mills, Siemens Healthineers, Abbott Laboratories

## Expert Testimony

None declared.

## Patents

None declared.
