# Correction to: Macrotroponin Complex as a Cause for Cardiac Troponin Increase after COVID-19 Vaccination and Infection

**DOI:** 10.1093/clinchem/hvad059

**Published:** 2023-05-09

Correction Notice for

Anda Bularga, Ellen Oskoui, Takeshi Fujisawa, Sara Jenks, Rachel Sutherland, Fred S Apple, Ola Hammarsten, Nicholas L Mills, Macrotroponin Complex as a Cause for Cardiac Troponin Increase after COVID-19 Vaccination and Infection, *Clinical Chemistry*, Volume 68, Issue 8, August 2022, Pages 1015–1019, https://doi.org/10.1093/clinchem/hvac100

In the originally published article three errors were identified as needing emendation.

In the section **Cardiac Troponin Immunoassay Interference**, the 3rd paragraph, the end of the 2nd sentence should read: “In patient 2, […] with a recovery of <1% and 17% respectively.”

instead of: “In patient 2, […] with a recovery of 3% in both cases.”

And Table 1, across the 25^th^ line marked *hs-cTnT recovery, %,* down the row for patient 2, the value should read: “17” instead of: “3”. And in Table 1, across the 28^th^ line, marked *hs-cTnT recovery, %,* down the row for patient 2, the value should read: “0.6” instead of: “3”.

These emendations have been made in the article. The author regrets this error.

